# Research on the Derated Power Data Identification Method of a Wind Turbine Based on a Multi-Gaussian–Discrete Joint Probability Model

**DOI:** 10.3390/s22228891

**Published:** 2022-11-17

**Authors:** Yuanchi Ma, Yongqian Liu, Zhiling Yang, Jie Yan, Tao Tao, David Infield

**Affiliations:** 1 State Key Laboratory of Alternate Electrical Power System with Renewable Energy Sources, North China Electric Power University, Beijing 102206, China; 2 Wind Energy and Control Centre, Department of Electronic and Electrical Engineering, University of Strathclyde, Glasgow G1 1XQ, UK

**Keywords:** wind turbine, SCADA data, derated power operation, multi-Gaussian–discrete joint probability model, EM algorithm

## Abstract

This paper focuses on how to identify normal, derated power and abnormal data in operation data, which is key to intelligent operation and maintenance applications such as wind turbine condition diagnosis and performance evaluation. Existing identification methods can distinguish normal data from the original data, but usually remove power curtailment data as outliers. A multi-Gaussian–discrete probability distribution model was used to characterize the joint probability distribution of wind speed and power from wind turbine SCADA data, taking the derated power of the wind turbine as a hidden random variable. The maximum expectation algorithm (EM), an iterative algorithm derived from model parameters estimation, was applied to achieve the maximum likelihood estimation of the proposed probability model. According to the posterior probability of the wind-power scatter points, the normal, derated power and abnormal data in the wind turbine SCADA data were identified. The validity of the proposed method was verified by three wind turbine operational data sets with different distribution characteristics. The results are that the proposed method has a degree of universality with regard to derated power operational data with different distribution characteristics, and in particular, it is able to identify the operating data with clustered distribution effectively.

## 1. Introduction

Wind power has achieved large-scale development and utilization on a global scale and has become the most widely used and fastest-growing renewable energy source [1]. As an important part of the wind turbine, the Supervisory Control And Data Acquisition (SCADA) system provides detailed data on the operating status of the wind turbine [2,3,4]. In recent years, the wind power industry has developed rapidly, and wind farms have accumulated a large quantity of operational data. Such data are indispensable for wind turbine state assessment and wind power prediction. In real time, they are also important for power system dispatch and to schedule any wind farm curtailment or derating.

Generally, wind turbine power curves exhibit a degree of scatter, reflecting measurement uncertainty in both wind speed and power. In addition, a certain amount of abnormal data usually exists in the actual measured operational data of the wind farm, which complicates interpretation such as determination of wind turbine operating state or wind power prediction. There are many factors affecting the quality of operational data, such as the measurement error of the sensor itself, poor measurement accuracy caused by a poor operating environment, data storage and transmission error, wind turbine performance failure, and importantly, the operating of a wind turbine in a derated power state. It is useful to divide abnormal operational data into two categories: often extensive data generated by the turbine under derated control; and a generally smaller amount of outlier data that deviate from the main data distribution, due to averaging over state transitions or some other sporadic factors [5,6,7]. However, there is no current SCADA parameter indicating whether it is derated or for the max power limit for a wind turbine. Therefore, how to identify a derated operation from within the turbine operational data and how to eliminate outliers are important research activities in the field of wind power.

Outlier detection is widely used in the field of wind power, and related research institutions have carried out significant research with useful results. There are many statistical methods for detecting outliers, which can be roughly divided into the following five categories: distribution-based outlier detection, depth-based outlier detection, cluster-based outlier detection, distance-based outlier detection, and outlier detection of density methods [8,9,10,11,12].

Distribution-based statistical outlier detection makes use of a fitted probability distribution for a given data set and identifies data that are far from this as outliers. The distribution-based outlier detection method is widely used in the wind power field. In [13], a mathematical model based on the quartile algorithm was used to identify the anomalous data. For the cases of a small amount of missing data, or in contrast, continuous missing blocks of data, the wind farm output correlation and multi-point cubic spline difference are used respectively for interpolation. The method reconstructs the missing data. In [14], the time series characteristics of bad data were identified, and a segmentation judgment method was applied. Any abnormal data are reconstructed based on the relationship between wind power output and the data characteristics of the wind farm itself. In [15], a joint probability model method based on the Copula function was proposed. By using the Copula function, a complex nonlinear multivariate relationship between parameters can be obtained based on the univariate marginal distribution of the data set. The significant outliers are then eliminated by examination of the derived joint probability model. In [16], an optimal intra-group variance algorithm for power curve analysis was proposed. This algorithm changes the dependence of traditional analysis methods on multi-dimensional data. It only needs to analyze wind speed and power and can identify the normal power generation status of the turbine. In [17], based on the analysis of the wind turbine-power abnormal operation data characteristics of wind turbines, the anomalous data are divided into four types: the bottom of the curve, the middle and upper stacking anomaly data, and the dispersive anomaly data around the curve. An anomalous data identification and cleaning process based on the combination of the change point grouping method and quartile method were proposed. This method can effectively identify four types of abnormal data, and the process is reasonable and the cleaning effect is good. The above distributed outlier detection methods can quickly and efficiently find outliers in the case of a known data set distribution. However, this method relies on the global distribution of a given data set and does not apply to situations where the high-dimensional data set and data set distribution are unknown.

In practice, most the operational data do not fully conform to a specific data model distribution [18,19]. To improve the distribution-based outlier detection method, the depth-based outlier detection method was created. This method assigns each data object a depth value and maps data objects to corresponding layers of a 2D space by the assigned depth values. Data objects on a shallow layer are more likely to be outliers than those on a deep layer. However, in practical applications, the existing depth-based outlier detection method is only effective in processing data in two-dimensional and three-dimensional space [20].

Cluster-based outlier detection divides the data set into clusters according to data features and identifies data points that are far away from any cluster as outliers. In [21], based on the k-means clustering, data stream concept drifting and existing outlier detection algorithm, a dynamic outlier detection algorithm was proposed. During the running of the algorithm, the sliding window size is adjusted adaptively according to the data flow concept drift detection result, and the cluster structure in the data set can be effectively found while determining the outliers. Cluster-based outlier detection can identify outliers in a fast and timely manner. The detection of outliers is very sensitive to the clustering algorithm used; consequently, the clustering algorithm must be selected with care.

In order to improve the above-mentioned outlier detection method, researchers have proposed a distance-based outlier detection method. This approach assesses whether the distance of most data points in the data set from the target point is greater than the user-defined distance threshold, and if so, the target point is considered to be an outlier [22,23]. In [24], a fast distance-based data outlier detection algorithm was proposed. The algorithm uses the sliding window model to process the data stream and uses the vector inner product inequality to reduce the branch. The distance-based outlier detection method is widely used because of its simplicity and efficiency. However, since the method uses the global threshold and does not consider the local density change, only global outliers can be detected, and local outliers cannot be detected.

The density-based outlier detection method is built on the distance-based outlier detection method, and it determines an outlier based on the field of the data point. It is able to accurately find outliers with uneven data distribution. In [25], in order to improve the efficiency of the existing density-based outlier detection algorithms, an outlier detection algorithm based on local density, LDBO, was introduced. The concept of strong k nearest neighbor and weak k near point was introduced. By analyzing the outlier correlation of adjacent data points, individual data points are treated differently. A data point outlier pre-judgment strategy was proposed to effectively improve the efficiency of the outlier detection algorithm for data distribution anomalies. The density-based outlier detection method can solve the problem of local outlier detection well, but there are still problems of high complexity and parameter selection.

The above-mentioned outlier detection methods each have advantages and disadvantages, and their scope of application is different. To overcome the problems caused by a single method, the current research mostly adopts a mixture of two or more methods. There are outlier detection methods based on distribution and clustering. In [26], based on the analysis of the data characteristics of the identified wind outliers, the outlier data combination detection model based on quartile method and k-means clustering analysis was proposed. The model does not rely on the normal data set for training and learning. It has strong automated processing capability and versatility, but the k value of the method is more complex and has a greater impact on the data processing results. There are outlier detection methods based on distribution, clustering and density mixing. In [27,28], two quartile algorithms were used to eliminate sparse outliers, and then the DBSCAN (Density-Based Spatial Clustering of Applications with Noise) algorithm was used to eliminate stacked outliers. The method does not need to input the number of clusters, and it has high accuracy and universality. However, when the spatial clustering density is not uniform and the cluster spacing difference is very large, the clustering quality is poor, and the outliers caused by the derated power are directly eliminated.

Instead of removing power curtailment data as outliers, our goal is to identify derated power operation states and eliminate outliers in wind turbine operating data that clearly deviate from the main trend, which contains several derated power operation states. The difficulty of the problem lies in the fact that there are data points generated by several turbines operating at several reduced power states within the data set. Not only outliers but also power curtailment data points are far from the power curve. Simply assessing distance from the power curve will not help here. Since there are more than one derated power states in the operational data, we cannot directly determine whether a specific data point belongs to the outliers based on the distance from the power curve. Therefore, in this paper, a method for detecting outliers resulting from derated power operation is proposed.

The method converts the outlier detection problem of the wind turbine with derated power operation into a mixed probability distribution model by introducing reasonable assumptions. The K-means clustering algorithm is used to initialize the parameters of the mixed probability model. Then, the expectation maximization (EM) algorithm is used to derive the updated expression for the model parameters. The logarithm likelihood function is maximized by an iterative method to obtain the optimal model parameters. Finally, the processing of the outliers of the wind turbine data in the power-reduced state is realized by calculating the posterior probability of the sample. The method proposed in this paper can quickly and efficiently identify the degrees of derating in the operational data, distinguish between normal operational data and several different degrees of derated power data, and eliminate outlier data in each data type to improve the quality of wind turbine operational data. The description of this approach is in four parts. The first section above introduced the research background and research status of the wind derated power operation data outlier detection method. The second section introduces the outlier detection model of the wind turbine derated power operation data. In the third section, real operating data of wind turbines located in North China are used to verify the proposed method. Finally, the conclusions are summarized.

## 2. Wind Turbine Derated Power Operation Data Outlier Detection Model

### 2.1. Modeling of Derated Power Operation Data Outlier Detection

**Figure 1:**
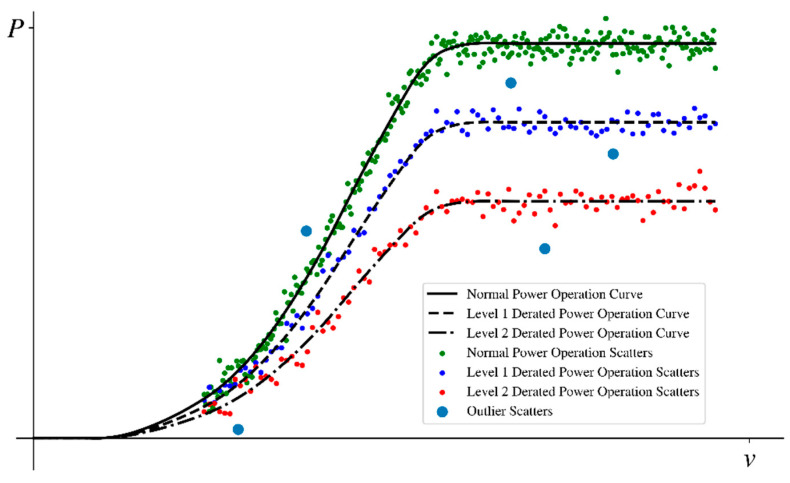
Schematic diagram of derated power operation status and outliers.

The method first identifies derated power levels contained in the operational data and then divides the data accordingly for derated power. Finally, the outliers are removed from the operational data corresponding to each type of derated power state. The notion of derated power levels and outliers are shown as Figure 1.

In keeping with statistical notation, random variables X,Y,Z represent the wind turbine output power, nacelle wind speed, and derated power state, respectively. Among them, X,Y are observable random variables. Z is a latent random variable and cannot be directly observed from the sample. From the SCADA system of the wind turbine, it is usually easy to observe a sample set of turbine output power and wind speed pairs $\left\{\left(x^{\left(1\right)},y^{\left(1\right)}\right),\ldots ,\left(x^{\left(m\right)},y^{\left(m\right)}\right)\right\}.$ Among them, $x^{\left(i\right)}\;\mathrm{and}\;y^{\left(i\right)}$ represent the *i*th output power and cabin wind speed samples in the data set, respectively. We need to establish the posterior probability distribution $p_{Z|XY}(z^{\left(i\right)}|x^{\left(i\right)},y^{\left(i\right)})$ of the output power X and the nacelle wind speed Y according to the sample data set Z, to identify the power limit of the sample $\left(x^{\left(i\right)},y^{\left(i\right)}\right)$, and further remove the outliers from the sample set whose posterior probability value is too low. In order to achieve this goal, a mixed probability density distribution $p_{XY}\left(x,y\right)$ with a derated power state Z as a latent random variable is firstly established.

### 2.2. Mixed Probability Density Distribution Model

In order to establish the mixed probability density distribution model $p_{XY}\left(x,y\right)$, the wind turbine derated power assumption and the derated power operation output assumption are introduced.

Power-limited state assumption: the derated power state of the turbine can be expressed in a limited state, that is, the wind turbine derated power state Z can take K different values. They respectively correspond to the normal operating state of the turbine and the K−1 derated power operating status with different derated power levels.

Power-limited state output assumption: the output of the wind turbine in a derated power operation state can be expressed as the theoretical output power multiplied by the corresponding derated power degree coefficient. That is, in a derated power state, the output power of the wind turbine can be expressed as $P_{k}\left(v\right)=\alpha_{k}f\left(v\right)$, where P=f(v) is a function of the wind turbine theoretical power curve, and v represents the incoming wind speed. $\alpha_{k}$ is the power limit coefficient corresponding to the *k*th derated power state, $\alpha_{k}\in \left[0,1\right]$, the smaller the value of $\alpha_{k}$ is, the greater the power limit of the unit is represented. The closer the value of $\alpha_{k}$ is to 1, the closer the unit state is to the normal power generation state. Here, the normal operating state can be regarded as a special derated power state in which the derated power degree coefficient $\alpha_{k}$ takes a value of 1.

In order to simplify the modeling process, we use the equal-width discrete method to discretize the wind speed data and divide the wind speed distribution interval into J equal parts. The spacing of each part is the same, and the median value of each wind speed interval represents the interval wind speed. It is further assumed that the discretized wind speed obeys the multinoulli distribution, i.e., Y∼Multinoulli(ψ), where vector ψ is the distribution parameter of the polynomial distribution and the *j*th element of the vector ψ satisfies $\psi_{j}\geq 0$, $\sum_{j=1}^{J}\psi_{j}=1$ and $p_{Y}\left(j\right)=\psi_{j}.$ It can be seen that the probability is$\;p_{Y}\left(y^{\left(i\right)}\right)=\sum_{j=1}^{J}I\left\{y^{\left(i\right)}\in V_{j}\right\}\cdot \psi_{j}$, when the wind speed is $y^{\left(i\right)}$, where I{⋅} represents the indication function. If the expression in the braces is true, the function value is 1; otherwise, the function value is 0; $I\left\{y^{\left(i\right)}\in v_{j}\right\}$ indicates whether the wind speed value $y^{\left(i\right)}$ corresponding to the sample i belongs to the discretized *j*th wind speed interval $V_{j}$.

The turbine’s derated power state Z cannot be directly observed; it is thus a latent random variable. It is assumed that the power-limited state also obeys the multinoulli distributions, that is, Z∼Multinoulli(ϕ), where the *k*th element of the vector ϕ satisfies $\phi_{k}\geq 0$, $\sum_{k=1}^{m}\phi_{k}=1$ and $p_{Z}\left(k\right)=\phi_{k}$.

Assume that under a given wind speed and derated power state, the turbine output power obeys a Gaussian distribution, i.e., $X|Y=y^{\left(i\right)},Z=k∼N(\mu_{k}\left(y_{\left(i\right)}\right),\;\sigma_{k}\left(y^{\left(i\right)}\right)$, where $\mu_{k}\left(y_{\left(i\right)}\right)$ and $\sigma_{k}\left(y^{\left(i\right)}\right)$ represent the mean and standard deviation of the Gaussian distribution at a given wind speed $y^{\left(i\right)}$ and a derated power k, respectively. According to the assumption of the derated power operation output of the wind turbine, the mean of the Gaussian distribution can be expressed as $\mu_{k}\left(y^{\left(i\right)}\right)=\alpha_{k}f\left(y^{\left(i\right)}\right)$; the standard deviation $\sigma_{k}\left(y^{\left(i\right)}\right)=\sum_{j}^{J}I\left\{y^{\left(i\right)}\in V_{j}\right\}\cdot \sigma_{jk}$, where $\alpha_{k}$ is the derated power coefficient corresponding to the *k*th derated power state, and $\sigma_{jk}$ is the standard deviation of the wind speed $y^{\left(i\right)}$ in the wind speed interval $V_{j}$, and the derated power degree is taken as k.

Furthermore, the wind speed Y and the derated power state Z are independent of each other; thus, $p_{Y|Z}\left(y|z\right)=p_{Y}\left(y\right)$.

According to the above assumptions, the mixed probability density distribution $p_{XY}\left(x,y\right)$ can be expressed as follows according to the conditional probability and the full probability formula:

(1) $$\begin{array}{c} \begin{array}{ll} p_{XY}\left(x^{\left(i\right)},y^{\left(i\right)}\right) & =\sum_{z^{\left(i\right)}}p_{XYZ}\left(x^{\left(i\right)},y^{\left(i\right)},z^{\left(i\right)}\right)\\ & =\sum_{z^{\left(i\right)}}p_{X|YZ}\left(x^{\left(i\right)}|y^{\left(i\right)},z^{\left(i\right)}\right)p_{Y|Z}\left(y^{\left(i\right)}|z^{\left(i\right)}\right)p_{Z}\left(z^{\left(i\right)}\right)\\ & =\sum_{z^{\left(i\right)}}p_{X|YZ}\left(x^{\left(i\right)}|y^{\left(i\right)},z^{\left(i\right)}\right)p_{Y}\left(y^{\left(i\right)}\right)p_{Z}\left(z^{\left(i\right)}\right) \end{array} \end{array}$$

Among them, $p_{X|YZ}\left(x^{\left(i\right)}|y^{\left(i\right)},z^{\left(i\right)}\right)$ indicates that under the condition that the power-limited state Z is $z^{\left(i\right)}$ and the wind speed random variable Y takes $y^{\left(i\right)}$, the conditional probability of the unit output power X can be calculated by Equation (2).

(2) $$\begin{array}{c} p_{X|YZ}\left(x^{\left(i\right)}|y^{\left(i\right)},k\right)=\frac{1}{\sqrt{2\pi }\sigma_{k}\left(y^{\left(i\right)}\right)}\exp\left(-\frac{1}{2\sigma_{k}^{2}\left(y^{\left(i\right)}\right)}{\left(x^{\left(i\right)}-\alpha_{k}f\left(y^{\left(i\right)}\right)\right)}^{2}\right) \end{array}$$

**Figure 2:**
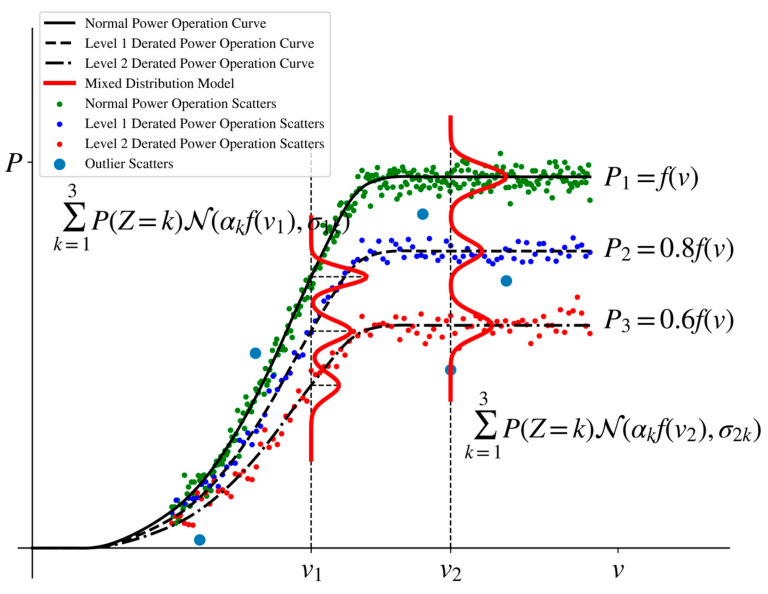
Schematic diagram of the mixed probability distribution model.

Figure 2 shows the joint probability distribution of wind speed and power in a derated power operation state of a wind turbine in this paper. If the above distribution function parameters $\alpha_{k},\sigma_{jk},\psi_{j},\phi_{k}$ are obtained, the probability values of the items in Equation (1) can be obtained. As shown in Equation (3), we can calculate the posterior probability $p_{Z|XY}(k|x^{\left(i\right)},y^{\left(i\right)})$ of each sample i under each derated power state k according to the Bayesian formula and then calculate the power-limited state to which the sample i belongs $c^{\left(i\right)}=\arg\underset{k}{\max}p_{Z|XY}(k|x^{\left(i\right)},y^{\left(i\right)})$. Finally, for each power-limited state k, the set of samples whose posterior probability is lower than the threshold $\left\{(x^{\left(i\right)},y^{\left(i\right)})|c^{\left(i\right)}=k,p_{Z|XY}\left(k|x^{\left(i\right)},y^{\left(i\right)}\right)<\theta \right\}$ is marked as outlier data to achieve outlier detection of wind turbine derated power operation data.

(3) $$\begin{array}{c} p_{Z|XY}(k|x^{\left(i\right)},y^{\left(i\right)})=\frac{p_{X|YZ}\left(x^{\left(i\right)}|y^{\left(i\right)},k;\alpha ,\sigma \right)p_{Y}\left(y^{\left(i\right)};\psi \right)p_{Z}\left(k;\phi \right)}{\sum_{k=1}^{K}p_{X|YZ}\left(x^{\left(i\right)}|y^{\left(i\right)},k;\alpha ,\sigma \right)p_{Y}\left(y^{\left(i\right)};\psi \right)p_{Z}\left(k;\phi \right)} \end{array}$$

To estimate the model parameters, a log-likelihood function can be written, as shown in Equation (4). Since Z is a latent random variable and cannot be directly observed, it is difficult to directly maximize the log-likelihood function (3) to solve the parameters. We turn to the idea of the EM algorithm to solve the problem.

(4) $$\begin{array}{c} ℒ\left(\alpha ,\sigma ,\psi ,\phi \right)=\sum_{i}\log p_{XY}\left(x^{\left(i\right)},y^{\left(i\right)};\alpha ,\sigma ,\psi ,\phi \right) \end{array}$$

### 2.3. EM Algorithm Estimation Model Parameters

According to the idea of the EM algorithm, we do not directly solve the maximum value of the log-likelihood function, and instead go to the lower bound (E-step) of the log-likelihood function and then maximize the lower bound (M-step). We can find the model parameters that maximize the likelihood function by iteratively repeating the E-step and M-step. We firstly introduce the Jensen inequality.

Theorem. Let f be a convex function and let X be a random variable.

Then:

(5) $$\begin{array}{c} E\left[f\left(X\right)\right]\geq f\left(E\left[X\right]\right) \end{array}$$

Moreover, if f is strictly convex, then E[f(X)]=f(E[X]) holds true if and only if X=E[X] with probability 1 (i.e., if X is a constant).

According to Jensen’s inequality, the lower bound of the log-likelihood function can be obtained:

(6) $$\begin{array}{c} \begin{array}{ll} ℒ\left(\alpha ,\sigma ,\psi ,\phi \right) & =\sum_{i}\log p_{XY}\left(x^{\left(i\right)},y^{\left(i\right)};\alpha ,\sigma ,\psi ,\phi \right)\\ & =\sum_{i}\log\sum_{z^{\left(i\right)}}p_{XYZ}\left(x^{\left(i\right)},y^{\left(i\right)},z^{\left(i\right)}\right)\\ & =\sum_{i}\log\sum_{z^{\left(i\right)}}Q_{i}\left(z^{\left(i\right)}\right)\frac{p_{XYZ}\left(x^{\left(i\right)},y^{\left(i\right)},z^{\left(i\right)}\right)}{Q_{i}\left(z^{\left(i\right)}\right)}\\ & \geq \sum_{i}\sum_{z^{\left(i\right)}}Q_{i}\left(z^{\left(i\right)}\right)\log\frac{p_{XYZ}\left(x^{\left(i\right)},y^{\left(i\right)},z^{\left(i\right)}\right)}{Q_{i}\left(z^{\left(i\right)}\right)} \end{array} \end{array}$$

Among them, ℒ(α,σ,ψ,ϕ) is a log-likelihood function of the mixed probability model; Q represents a certain distribution, and the condition that the inequality takes the equal sign is that $\frac{p_{XYZ}\left(x^{\left(i\right)},y^{\left(i\right)},z^{\left(i\right)}\right)}{Q_{i}\left(z^{\left(i\right)}\right)}$ is a constant. According to $\sum_{z^{\left(i\right)}}Q_{i}\left(z^{\left(i\right)}\right)=1$, you can obtain:

(7) $$\begin{array}{c} \begin{array}{ll} Q_{i}\left(z^{\left(i\right)}\right) & =\frac{p_{XYZ}\left(x^{\left(i\right)},y^{\left(i\right)},z^{\left(i\right)}\right)}{\sum_{z^{\left(i\right)}}p_{XYZ}\left(x^{\left(i\right)},y^{\left(i\right)},z^{\left(i\right)}\right)}\\ & =p_{Z|XY}\left(z^{\left(i\right)}|x^{\left(i\right)},y^{\left(i\right)}\right) \end{array} \end{array}$$

Let $w_{k}^{\left(i\right)}=Q_{i}\left(k\right)=p_{Z|XY}(k|x^{\left(i\right)},y^{\left(i\right)})$. According to the Bayesian formula, you can obtain:

(8) $$\begin{array}{c} w_{k}^{\left(i\right)}=\frac{p_{X|YZ}\left(x^{\left(i\right)}|y^{\left(i\right)},k;\alpha ,\sigma \right)p_{Y}\left(y^{\left(i\right)};\psi \right)p_{Z}\left(k;\phi \right)}{\sum_{k=1}^{K}p_{X|YZ}\left(x^{\left(i\right)}|y^{\left(i\right)},k;\alpha ,\sigma \right)p_{Y}\left(y^{\left(i\right)};\psi \right)p_{Z}\left(k;\phi \right)} \end{array}$$

Let ℓ(α,σ,ψ,ϕ) take the right side of the inequality of Equation (5) as the lower bound of the log-likelihood function, then ℓ(α,σ,ψ,ϕ) can be expressed as:

(9) $$\begin{array}{c} \ell \left(\alpha ,\sigma ,\psi ,\phi \right)=\sum_{i=1}^{m}\sum_{k=1}^{K}w_{k}^{\left(i\right)}\log\frac{\frac{1}{\sqrt{2\pi }\sigma_{k}\left(y^{\left(i\right)}\right)}\exp[-\frac{1}{2\sigma_{k}^{2}\left(y^{\left(i\right)}\right)}\left(x^{\left(i\right)}-\alpha_{k}f{\left(y^{\left(i\right)}\right)}^{2}\right]\psi \left(y^{\left(i\right)}\right)\phi_{k}}{w_{k}^{\left(i\right)}} \end{array}$$

where $\psi \left(y^{\left(i\right)}\right)=p_{Y}\left(y^{\left(i\right)}\right)=\sum_{j=1}^{J}I\left(y^{\left(i\right)}\in V_{j}\right)\cdot \psi_{j}$, $\sigma_{k}\left(y^{\left(i\right)}\right)=\sum_{j=1}^{J}I\left(y^{\left(i\right)}\in V_{j}\right)\cdot \sigma_{jk}$.

After obtaining the lower bound ℓ(α,σ,ψ,ϕ) of the log-likelihood function, we can obtain the partial derivative of the lower bound on the parameters α,σ,ψ,ϕ. Then, let the partial derivative equal zero, and obtain the model parameters by maximizing the lower bound ℓ(α,σ,ψ,ϕ) of the log-likelihood function.

Find the partial derivative of ℓ to $\alpha_{q}$

(10) $$\begin{array}{c} \frac{\partial \ell }{\partial \alpha_{q}}=\sum_{i=1}^{m}w_{q}^{\left(i\right)}\frac{[x^{\left(i\right)}-\alpha_{q}f\left(y^{\left(i\right)})\right]f\left(y^{\left(i\right)}\right)}{\sigma_{q}\left(y^{\left(i\right)}\right)} \end{array}$$

Let the above formula be equal to zero, and we find:

(11) $$\begin{array}{c} \alpha_{q}=\frac{\sum_{i=1}^{m}w_{q}^{\left(i\right)}x^{\left(i\right)}f\left(y^{\left(i\right)}\right)}{\sum_{i=1}^{m}w_{q}^{\left(i\right)}f^{2}\left(y^{\left(i\right)}\right)} \end{array}$$

Next, we find the partial derivative of ℓ on $\sigma_{pq}$:

(12) $$\begin{array}{c} \begin{array}{ll} \frac{\partial \ell }{\partial \sigma_{pq}} & =\sum_{i=1}^{m}\frac{\partial }{\partial \sigma_{pq}}w_{q}^{\left(i\right)}I\left(y^{\left(i\right)}\in V_{p}\right)[-\log\sigma_{pq}-\frac{1}{2\sigma_{pq}^{2}}\left(x^{\left(i\right)}-\alpha_{q}f{\left(y^{\left(i\right)}\right)}^{2}\right]\\ & =\sum_{i}^{m}w_{q}^{\left(i\right)}I\left(y^{\left(i\right)}\in V_{p}\right)\left[-\frac{1}{\sigma_{pq}}+\frac{1}{\sigma_{pq}^{3}}{\left(x^{\left(i\right)}-\alpha_{q}f\left(y^{\left(i\right)}\right)\right)}^{2}\right] \end{array} \end{array}$$

Let the above formula be equal to zero, and we find:

(13) $$\begin{array}{c} \sigma_{pq}^{2}=\frac{\sum_{i=1}^{m}w_{q}^{\left(i\right)}I\left(y^{\left(i\right)}\in V_{p}\right)(x^{\left(i\right)}-\alpha_{q}f{\left(y^{\left(i\right)}\right)}^{2}}{\sum_{i=1}^{m}w_{q}^{\left(i\right)}I\left(y^{\left(i\right)}=p\right)} \end{array}$$

Find the partial derivative of ℓ on $\phi_{q}$. $\sum_{k=1}^{K}\phi_{k}=1$. Using the Lagrangian multiplier method, we find the partial derivative of $\ell +\lambda \left(\sum_{k=1}^{K}\phi_{k}-1\right)$ on $\phi_{q}$ and λ.

(14) $$\begin{array}{c} \frac{\partial }{\partial \phi_{q}}\left(\ell +\lambda \left(\sum_{k=1}^{K}\phi_{k}-1\right)\right)=\sum_{i}^{m}\frac{w_{q}^{\left(i\right)}}{\phi_{q}}+\lambda \end{array}$$

(15) $$\begin{array}{c} \frac{\partial }{\partial \lambda }\left(\ell +\lambda \left(\sum_{k=1}^{K}\phi_{k}-1\right)\right)=\sum_{k=1}^{K}\phi_{k}-1 \end{array}$$

Let the upper two formulas be equal to zero. Combine these two formulas and we can obtain the solution:

(16) $$\begin{array}{c} \phi_{q}=\frac{1}{m}\sum_{i=1}^{m}w_{q}^{\left(i\right)} \end{array}$$

In the same way, $\sum_{j=1}^{J}\psi_{j}=1$. Using the Lagrangian multiplier method, we find the partial derivative of $\ell +\gamma \left(\sum_{j=1}^{J}\psi_{j}-1\right)$ on $\psi_{p}$ and γ.

(17) $$\begin{array}{c} \begin{array}{ll} \frac{\partial }{\partial \psi_{p}}\left(\ell +\gamma \left(\sum_{j}^{J}\psi_{j}-1\right)\right) & =\frac{\partial }{\partial \psi_{p}}\left(\sum_{i=1}^{m}\sum_{k=1}^{K}w_{k}^{\left(i\right)}I\left(y^{\left(i\right)}\in V_{p}\right)\cdot \log\psi_{p}+\gamma \left(\sum_{j=1}^{J}\psi_{j}-1\right)\right)\\ & =\sum_{i=1}^{m}\sum_{k=1}^{K}\frac{w_{k}^{\left(i\right)}I\left(y^{\left(i\right)}\in V_{p}\right)}{\psi_{p}}+\lambda \end{array} \end{array}$$

(18) $$\begin{array}{c} \frac{\partial }{\partial \gamma }\left(\ell +\gamma \left(\sum_{j}^{J}\psi_{j}-1\right)\right)=\sum_{j}^{J}\psi_{j}-1 \end{array}$$

Let the upper two formulas be equal to zero. Combine these two formulas and we can obtain the solution:

(19) $$\begin{array}{c} \psi_{p}=\frac{\sum_{i=1}^{m}\sum_{k=1}^{K}I\left(y^{\left(i\right)}\in V_{p}\right)w_{k}^{\left(i\right)}}{m} \end{array}$$

Thus far, we have derived two main processes, E-step and M-step, in the EM algorithm. In the E-step, according to the initialized parameters, we can calculate $w_{k}^{\left(i\right)}$ according to Formula (8). In the M-step, the parameters α,σ,ψ,ϕ are updated according to Equations (11), (13), (16) and (19); the E-step and M-step are repeated repeatedly until convergence. Then, we can find the model parameters.

Convergence is guaranteed by the EM algorithm. Hence, we will no longer discuss the proof process in this paper. However, the EM algorithm can only converge to the local optimum, and the result is greatly affected by the initial value. Here, we will give the identification method of the optimal derated power level number K and the initialization method of other parameters of the model to help the EM algorithm to quickly and stably converge.

### 2.4. Optimal Derated Power Level Identification

The parameters of the wind turbine derated power operation data anomaly detection model proposed in this paper include: the number of derated power levels K; the number of discretized wind speed intervals J; the derated power coefficient $\alpha =\left[\alpha_{1},\ldots ,\alpha_{K}\right]$, the power limit distribution parameter $\phi =\left[\phi_{1},\ldots ,\phi_{K}\right]$, the discretized wind speed probability distribution parameter $\psi =\left[\psi_{1},\ldots ,\psi_{J}\right]$ and the mean parameter of the Gaussian distribution $\mu_{jk}$ and the variance parameter $\sigma_{jk}^{2}$. We firstly determine the optimal derated power level number K.

Inspired by the k-means clustering algorithm, we firstly give the derated power level number K and the discretized wind speed interval number J and random initialization $\alpha =\left[\alpha_{1},\ldots ,\alpha_{K}\right]$, $\psi =\left[\psi_{1},\ldots ,\psi_{K}\right]$, $\phi =\left[\phi_{1},\ldots ,\phi_{K}\right]$, $\mu_{jk}$ and $\sigma_{jk}^{2}$. For each sample i, we calculate the distance $d_{k}^{\left(i\right)}=\left|x^{\left(i\right)}-\alpha_{k}f\left(y^{\left(i\right)}\right)\right|$ from $\left(x^{\left(i\right)},y^{\left(i\right)}\right)$ to each of the derated power output curves. We find the derated power state corresponding to the curve with the smallest distance from the sample i to each of the derated power state running curves as the power limit state of the sample $\left(x^{\left(i\right)},y^{\left(i\right)}\right)$, denoted as $c^{\left(i\right)}=\arg\underset{k}{\min}\;d_{k}^{\left(i\right)}$. The distance of the sample i from the corresponding derated power running curve is recorded as $d^{\left(i\right)}=\underset{k}{\min}\;d_{k}^{\left(i\right)}$. For the sample set of the same power state $\left\{\left(x^{\left(i\right)},y^{\left(i\right)}\right)|c^{\left(i\right)}=k\right\}$, we use the least squares fitting derated power operation curve $x=\alpha_{k}f\left(y\right)$ and update the corresponding derated power coefficient $\alpha_{k}$.

Let *K* take 2-8 in sequence and repeat the above steps several times. We calculate the average loss value of all samples $\sum_{i}\frac{d^{i}}{m}$ and take the average value of each loss. We use the mean as the vertical axis and the *K* value as the horizontal axis as the elbow curve. The *K* value corresponding to the position where the average loss function value has the largest decrease is taken as the optimal derated power level number.

### 2.5. Model Parameter Initialization Method

After obtaining the optimal power level number K and the power limit class $c^{\left(i\right)}$ corresponding to each sample, we can directly write the initialization expression of the parameters ψ,ϕ,μ,σ, as shown in Formula (20) to Formula (23).

(20) $$\begin{array}{c} \psi_{j}=\frac{\sum_{i=1}^{m}I\left\{y^{\left(i\right)}\in V_{j}\right\}}{m} \end{array}$$

(21) $$\begin{array}{c} \phi_{k}=\frac{\sum_{i=1}^{m}I\left\{c^{\left(i\right)}=k\right\}}{m} \end{array}$$

(22) $$\begin{array}{c} \mu_{jk}=\frac{\sum_{i=1}^{m}I\{c^{\left(i\right)}=k,y^{\left(i\right)}\in V_{j}\}x^{\left(i\right)}}{m} \end{array}$$

(23) $$\begin{array}{c} \sigma_{jk}^{2}=\frac{\sum_{i=1}^{m}I\left\{c^{\left(i\right)}=k,y^{\left(i\right)}\in V_{j}\right\}{\left(x^{\left(i\right)}-\mu_{jk}\right)}^{2}}{m} \end{array}$$

According to the derated power level $c^{\left(i\right)}$ corresponding to each sample, we can initially realize the division of the derated power data, but the result obtained by this process is not the optimal result, and based on the k-means clustering algorithm, it is also impossible to eliminate outliers in each of the derated power levels. However, we can use the parameters obtained in the above process as the initial values of the EM algorithm, so that the parameters of the final model can be stably converged to similar local best points.

Based on the above modeling process and parameter initialization process, the algorithm for the anomaly detection method of wind turbine derated power operation data proposed in this paper is as follows (Algorithm 1):
**Algorithm 1** Wind turbine derated power operation data outlier detectionRequire: the sample set of turbine output power and nacelle wind speed pair $\left\{\left(x^{\left(1\right)},y^{\left(1\right)}\right),\ldots ,\left(x^{\left(m\right)},y^{\left(m\right)}\right)\right\}$, discretized wind speed interval number J=50, wind turbine theoretical power curve function P=f(v), probability threshold θ. 1. Initialize the model parameters to obtain the optimal power level number K and the initial values of the mixed probability model parameters $\alpha^{\left(0\right)},\sigma^{\left(0\right)},\psi^{\left(0\right)},\phi^{\left(0\right)}$. 2. While α,σ,ψ,ϕ have no convergence. Do 3.    E-step: For each sample i and the derated power k,      calculate $w_{k}^{\left(i\right)}=\frac{p_{X|YZ}\left(x^{\left(i\right)}|y^{\left(i\right)},k;\alpha ,\sigma \right)p_{Y}\left(y^{\left(i\right)};\psi \right)p_{Z}\left(k;\phi \right)}{\sum_{k=1}^{K}p_{X|YZ}\left(x^{\left(i\right)}|y^{\left(i\right)},k;\alpha ,\sigma \right)p_{Y}\left(y^{\left(i\right)};\psi \right)p_{Z}\left(k;\phi \right)}$. 4.    M-step: Update parameters 5.        $\alpha_{q}=\frac{\sum_{i=1}^{m}w_{q}^{\left(i\right)}x^{\left(i\right)}f\left(y^{\left(i\right)}\right)}{\sum_{i=1}^{m}w_{q}^{\left(i\right)}f^{2}\left(y^{\left(i\right)}\right)}$ 6.        $\sigma_{pq}^{2}=\frac{\sum_{i=1}^{m}w_{q}^{\left(i\right)}I\left(y^{\left(i\right)}\in V_{p}\right)(x^{\left(i\right)}-\alpha_{q}f{\left(y^{\left(i\right)}\right)}^{2}}{\sum_{i=1}^{m}w_{q}^{\left(i\right)}I\left(y^{\left(i\right)}\in V_{p}\right)}$ 7.        $\phi_{q}=\frac{1}{m}\sum_{i=1}^{m}w_{q}^{\left(i\right)}$ 8.        $\psi_{p}=\frac{\sum_{i=1}^{m}\sum_{k=1}^{K}I\left(y^{\left(i\right)}\in V_{p}\right)w_{k}^{\left(i\right)}}{m}$ 9. End while. 10. Calculate the posterior probability $p_{Z|XY}(k|x^{\left(i\right)},y^{\left(i\right)})$ of each sample i under each derated power state k according to iterative parameters. 11. Calculate the derated power level $c^{\left(i\right)}=\arg\underset{k}{\max}\;p_{Z|XY}(k|x^{\left(i\right)},y^{\left(i\right)})$ to which the sample i belongs. 12. For each derated power k, the sample set whose posterior probability is lower than the threshold $\text{\{}(x^{\left(i\right)},y^{\left(i\right)})|c^{\left(i\right)}=k,p_{Z|XY}\left(k|x^{\left(i\right)},y^{\left(i\right)}\right)<\theta \}$ is marked as outlier data.

## 3. Analysis of the Case of Outlier Detection of Wind Power Turbine Derated Power Operation Data

SCADA data of a wind farm in North China are used to verify the effectiveness of the proposed outlier detection method for wind turbine derated power operation data. North China is a location where “wind curtailment” is common. Wind data from this area are suitable for studying data processing methods for derated power operation. In order to fully verify the method proposed in this paper, we select the operating data of three 2.5 MW direct-drive permanent-magnet synchronous generator wind turbines (#1, #2, #6) with different derated power levels on the wind farm. The basic information for these three wind turbines is shown in Table 1. The operating data of the SCADA system of the turbine were intercepted from 10:30 a.m. on 20 November 2014 to 10:10 a.m. on 19 January 2015 for analysis.

### 3.1. Case Analysis

**Figure 3:**
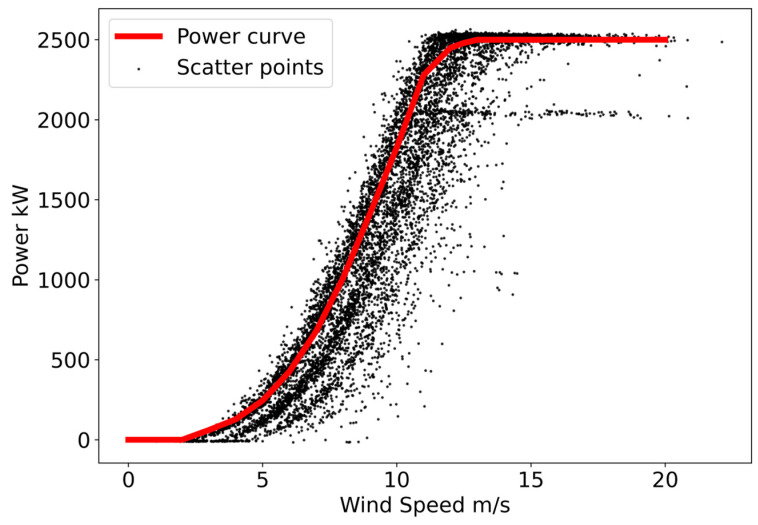
Wind speed and power scatter plot of turbine #2 and the manufacturer’s power curve that corrected for air density in the standard manner.

The method proposed in this paper will be described in a detailed way by taking the #2 turbine as an example. We plot the wind speed and power scatter plot of the turbine and the manufacturer’s power curve that corrected for air density in the standard manner, as shown in Figure 3. It can be seen from the figure that the power scatter plot of turbine #2 is approximately distributed over three clusters and that a large proportion of the points reflect derated power operation. The uppermost cluster of scatter points is closest to the manufacturer’s power curve, and the turbine can be considered here to be in normal operation. The following two clusters of scatter points correspond to different derated power states of the turbine. There are still some outliers in the scatter plot that deviate significantly from the main trends, which we have to identify and eliminate. Since there is more than one derated power state in the operational data, we cannot directly determine whether a specific data point belongs to the outliers based on the distance from the power curve. To do this, we need to identify and classify the derated power operating states of the operational data and then remove those outliers from the samples corresponding to each type of derated power state.

**Figure 4:**
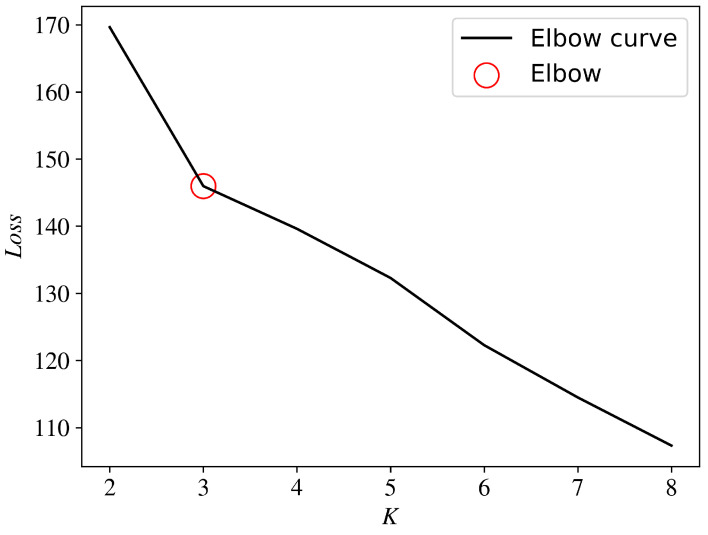
Elbow curve to determine the optimal derated power level.

In order to determine the optimal derated power level, the elbow curve is drawn according to the best derated power level identification method proposed in Section 2.4, as shown in Figure 4. It can be seen from the figure that the *K* value corresponding to the position where the average loss function value has the largest decrease is 3. It can be seen that this set of data contains one set of normal operating states and two different levels of derated power operating states, which is consistent with the results we observed directly from the data.

**Figure 5:**
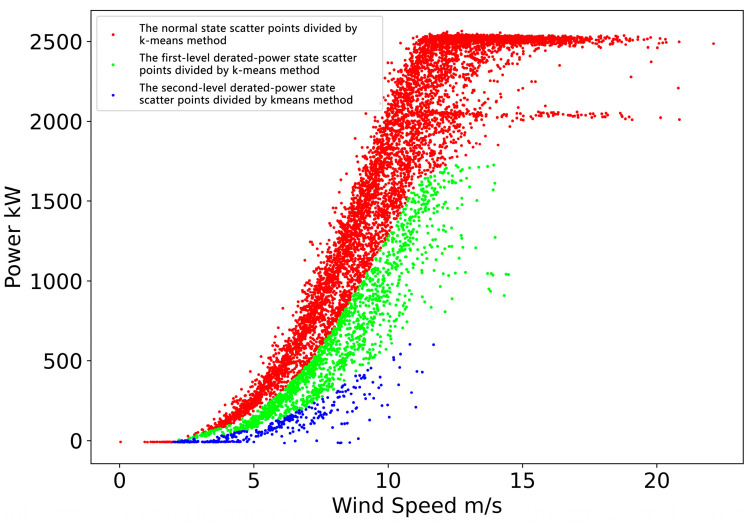
Operation data derated power state division result obtained by k-means method.

After the initialization step, we can obtain the preliminary operation data derated power state allocation, as shown in Figure 5. The result is obtained based on the distance of the scatter distance from each of the derated power curves. It can be seen from the figure that the scatter points of red, green and blue respectively represent the division of the normal operating state and the provisional first and second derated power levels given by the model. This step preliminarily realizes the division of the derated power level of the operational data, but it is incomplete. We will initialize the parameters of the probability model based on this result to achieve a more refined model.

**Figure 6:**
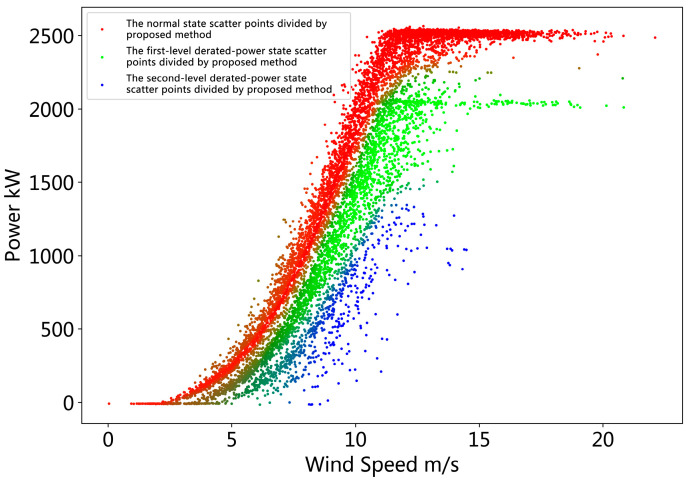
Operational data derated power state division result obtained by the proposed method.

According to the method proposed in Section 2.2, the mixed probability model is established, and the mixed probability model parameters are estimated by the EM algorithm according to the proposed method in Section 2.3. Then, the posterior probability $p_{Z|XY}(k|x^{\left(i\right)},y^{\left(i\right)})$ of each sample i under each derated power state k is calculated according to the Bayesian formula. Then, the probability value $c^{\left(i\right)}=\arg\underset{k}{\max}\;p_{Z|XY}(k|x^{\left(i\right)},y^{\left(i\right)})$ of the derated power level to which the sample i belongs is calculated to implement the division of the derated power state of the operating data. Figure 6 shows the results of the partitioning of the mixed probability model. We use different colors to represent the derated power state to which each sample belongs. The red points represent a higher probability that the sample is in normal operation. The green and blue points respectively represent a higher probability that the sample belongs to the derated power state 1 and the derated power state 2. Sample color between two derated power states will mix. This is because the probability that the sample belongs to these two power-limited states is very close. This reflects the uncertainty of the derated power state to which the sample belongs.

**Figure 7:**
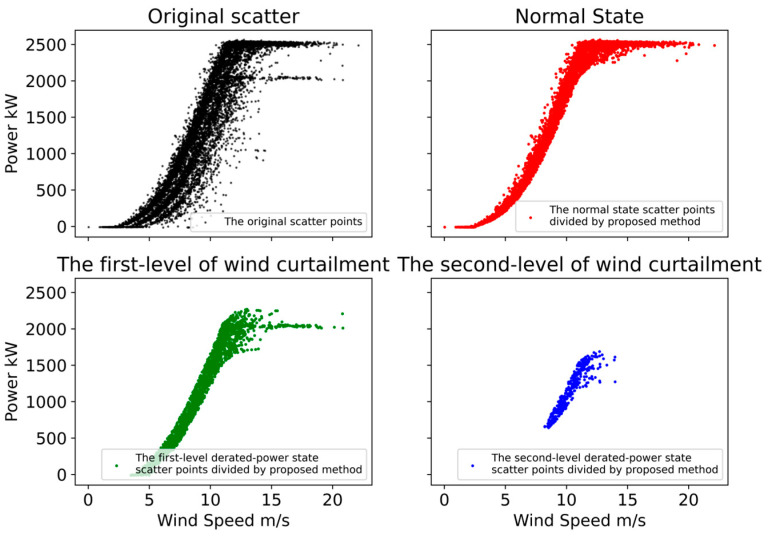
Operation data derated power state division and outliers elimination result.

For each derated power state k, we give a threshold θ=0.8. The sample set $\left\{(x^{\left(i\right)},y^{\left(i\right)})|c^{\left(i\right)}=k,p_{Z|XY}\left(k|x^{\left(i\right)},y^{\left(i\right)}\right)<\theta \right\}$ with the posterior probability below the threshold is eliminated to achieve outlier elimination. The result is shown in Figure 7. Compared with the results of k-means partitioning, the proposed method is more effective. In addition, it will be seen from the following cases that more flexible outlier detection can be achieved by giving different thresholds θ in the process of eliminating outliers.

### 3.2. Comparison of Outlier Detection Results of Wind Turbine Operation Data with Different Derated Power Levels

**Figure 8:**
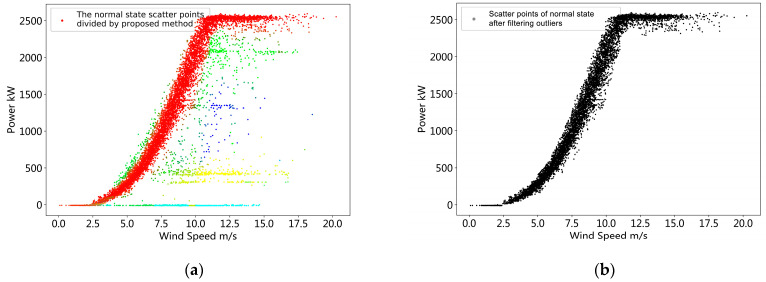
Operation data derated power state division and outlier elimination result of turbine #1. (**a**) The derated power state division result of turbine #1, in this case, five different colors represent five derated power levels in the dataset of turbine #1, and red points represents normal power operational state; (**b**) the outliers elimination result under normal operating conditions of turbine #1.

**Figure 9:**
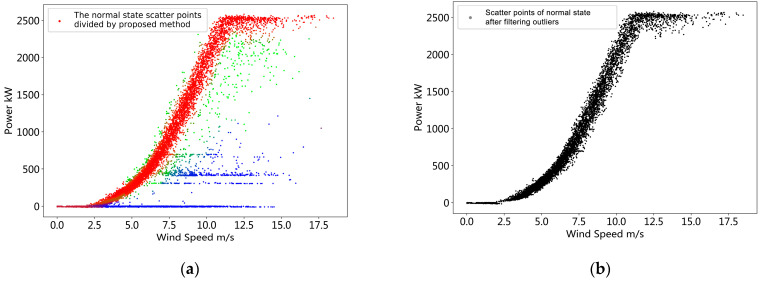
Operation data derated power state division and outlier elimination result of turbine #6. (**a**) The derated power state division result of turbine #6, in this case, three different colors represent three derated power levels in the dataset of turbine #6, and red points represents normal power operational state; (**b**) the outliers elimination result under normal operating conditions of turbine #6.

In order to further verify the effectiveness of the method, we performed the same steps as those in Section 3.1 for the operation data of turbine #1 and turbine #6. We obtained the derated power data degree division and outlier detection results of the operational data of the two turbines, as shown in Figure 8 and Figure 9. It can be seen from Figure 8a that the method proposed in this paper identifies that the operating data of turbine #1 contains five operating states, including normal operating conditions and four derated power operating states. From the results of the derated power state division, the derated power data are clearly separated from the normal state data, and the four derated power states also achieve good distinction. After the threshold value *θ* is set, the outliers corresponding to the respective operating states can be detected. Since there are less derated power data in the operation data of turbine #1, we will not display the operation data under each derated power state and only draw the results after removing the outliers in normal operation, as shown in Figure 8b.

Similarly, as can be seen from Figure 9a, the method proposed in this paper identifies that the operating data of turbine #6 contains three operating states, including normal operating conditions and two derated power operating states. From the results of the derated power state division, the derated power data and the normal state data achieve a good separation, but the identification of the two derated power states does not achieve the expected effect. The green data points are outliers from normal operation, while the blue data points contain two derated power states. However, this does not affect the outlier detection under normal operating conditions. It can be seen from Figure 9b that the good outlier detection effect is also achieved under the normal operation of turbine #6.

Comparing turbine #1 and turbine #6, we can see that the method proposed in this paper can adapt to different operating numbers of derated power levels and is more reliable in the detection of outliers.

### 3.3. Impact of Threshold on Outlier Detection Results

**Figure 10:**
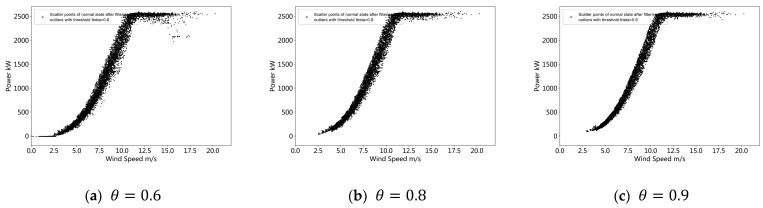
Impact of threshold *θ* on ourlier detection result.

The method proposed in this paper relies on the posterior probability value of the sample to detect the outliers. When the posterior probability of the sample is less than the given threshold, the sample is judged as an outlier. Therefore, if different thresholds are applied, different outliers will be identified. To investigate the effect of thresholds on outlier detection, the same turbine data are used with the same model but with different thresholds. We establish a mixed probability model for the operating data of turbine #1 and estimate the model parameters to obtain the posterior probability. The threshold values *θ* are set to 0.6, 0.8, and 0.9. The outlier detection results are shown in Figure 9. It can be observed from the figure that when the threshold is low (*θ* = 0.6), more sample data will be retained, and when the threshold is higher (*θ* = 0.9), less data will be retained. As the threshold gradually increases, the points in the lower probability density regions will be gradually removed, which is consistent with expectations. Therefore, the threshold can be flexibly selected according to the quality requirements of the operational data. Selecting a higher threshold results in fewer samples with outliers, and selecting a lower threshold preserves more raw data. In this case, selecting a threshold of 0.8 eliminates most outliers and preserves as much raw data as possible. Furthermore, from the comparison of Figure 10a–c, it can be found that changing the threshold *θ* over a larger range does not produce a huge change in the outcome. The result is still able to maintain the data that need to be retained, that is, the model is robust to the choice of threshold.

## 4. Conclusions

In this paper, we propose an outlier detection method suitable for wind turbines with derated power operation. The following conclusions can be drawn from the analysis of the example:

1. By introducing reasonable assumptions regarding derated power operation, a mixed probability distribution model of wind speed and power with derated power as an implicit random variable is established. Outlier detection under the derated power level of the wind turbine is realized. The method is based on statistical theory and the expectation maximization algorithm and has a sound theoretical basis.
2. A mixed probability model initialization method based on k-means clustering is established. This method can identify derated power levels in the data and provide the initial value of the parameter of the mixed probability model. It can also accelerate the convergence of model parameters, making the results more stable.
3. The example shows that the method can reliably detect outliers of the wind turbine under normal operating conditions and under different derated power states. The method can also distinguish different derated power state data and has certain robustness to the selection of model parameters.

## Figures and Tables

**Table 1:** Basic parameters of wind turbines.

Parameter	Value
Rated power/kW	2500
Power adjustment method	Variable pitch, variable speed
Number of blades	3
Rotor diameter/m	103
Hub height/m	80
Cut-in wind speed/(m/s)	3
Rated wind speed/(m/s)	11
Cut-out wind speed/(m/s)	25
Maximum wind speed/(m/s)	39.6
Extreme wind speed/(m/s)	55
Air density/kg/m^3^	1.0622

## Data Availability

Not applicable.
